# Air-breathing behavior, oxygen concentrations, and ROS defense in the swimbladders of two erythrinid fish, the facultative air-breathing jeju, and the non-air-breathing traira during normoxia, hypoxia and hyperoxia

**DOI:** 10.1007/s00360-017-1142-1

**Published:** 2018-01-03

**Authors:** Bernd Pelster, Chris M. Wood, Ellen Jung, Adalberto L. Val

**Affiliations:** 1 Institut für Zoologie, Leopold-Franzens-Universität Innsbruck, Technikerstr. 25, 6020 Innsbruck, Austria; 2 Center for Molecular Biosciences, University Innsbruck, Innsbruck, Austria; 3 Department of Zoology, University of British Columbia, Vancouver, BC V6T 1Z4 Canada; 4 Instituto Nacional de Pesquisas da Amazônia, Manaus, Brazil

**Keywords:** Air-breathing fish, ROS defense, Swimbladder, Hypoxia, Hyperoxia

## Abstract

The jeju *Hoplerythrinus unitaeniatus* and the traira *Hoplias* *malabaricus* are two neighboring genera from the family of erythrinid fish, both possessing a two-chambered physostomous swimbladder. In the jeju the anterior section of the posterior bladder is highly vascularized, and the swimbladder is used for aerial respiration; the traira, in turn, is a water-breather that uses the swimbladder as a buoyancy organ and not for aerial oxygen uptake. Measurement of swimbladder oxygen partial pressure (PO_2_) of fish kept at 26 °C in normoxic, hyperoxic (28–32 mg O_2_ L^− 1^) or hypoxic (1–1.5 mg O_2_ L^− 1^) water revealed constant values in traira swimbladder. Under normoxic conditions in the jeju swimbladder PO_2_ was higher than in traira, and the PO_2_ significantly increased under hyperoxic conditions, even in the absence of air breathing. In jeju, air-breathing activity increased significantly under hypoxic conditions. Hypoxic air-breathing activity was negatively correlated to swimbladder PO_2_, indicating that the swimbladder was intensely used for gas exchange under these conditions. In traira, the capacity of the ROS defense system, as assessed by measurement of activities of enzymes involved in ROS degradation and total glutathione (GSH + GSSG) concentration, was elevated after 4 h of hyperoxic and/or hypoxic exposure, although swimbladder PO_2_ was not affected. In jeju, experiencing a higher variability in swimbladder PO_2_ due to the air-breathing activity, only a reduced responsiveness of the ROS defense system to changing environmental PO_2_ was detected.

## Introduction

The oxygen concentration in the Amazon Basin is known to vary greatly, depending on season and the daily cycle (Val and Almeida-Val 1995; Muusze et al. 1998; Diaz and Breitburg 2009; Welker et al. 2013). To supplement oxygen demand during periods of aquatic hypoxia, many fish of the Amazon Basin rely on aquatic surface respiration or even use specific tissues or organs for aerial gas exchange (Val and Almeida-Val 1995). In water-breathing fish arterial PO_2_ typically is well below aerial PO_2_ (Gilmour and Perry 1994; Kristensen et al. 2010). Epithelia exposed to air in these air breathing organs therefore face much higher PO_2_ values than tissues of water-breathing fish. Depending on the air-breathing activity and the removal of oxygen from the engulfed air, also quite variable PO_2_ values may be encountered in air-breathing organs.

High oxygen concentrations and especially hyperoxic conditions result in the generation of reactive oxygen species (ROS), not only in mammals but also in fish. Exposure to hyperoxia caused oxidative stress in different tissues of goldfish (brain, liver, kidney, muscle), as evidenced by an increase in protein carbonylation, TBARS (a byproduct of lipid peroxidation) and/or lipid peroxides themselves (Lushchak and Bagnyukova 2006; Lushchak 2011). Lipid peroxidation appears to be one of the most rapid responses to hyperoxia and this stress signal may trigger antioxidant systems (Lushchak and Bagnyukova 2006). In the air-breathing fish *Heteropneustes fossilis*, 3 h of aerial exposure resulted in an increase in protein carbonylation, an increase in TBARS, and after 6 h, H_2_O_2_ concentration was elevated in brain tissue (Paital 2013). In muscle tissue, TBARS and H_2_O_2_ were elevated after 3 h of exposure and protein carbonylation after 6 h (Paital 2014). These results support the conclusion that air-exposed epithelia in fish face oxidative stress, and this does not only affect the air-exposed epithelia, it affects the whole organism. Indeed, in a recent study on erythrinid fishes, we demonstrated that there was a higher ROS defense capacity not only in the swimbladder tissue but also in the muscle tissue, of the facultative air-breathing jeju (*Hoplerythrinus unitaeniatus*) relative to its non-air-breathing close relative, the traira (*Hoplias malabaricus*) (Pelster et al. 2016). The jeju uses its swimbladder as an air-breathing organ, whereas the traira uses the swimbladder only as a buoyancy organ. In non-ventilated gas cavities such as the traira swimbladder, inert gases such as nitrogen accumulate (Piiper 1965), so that PO_2_ in traira swimbladder is expected to be much lower than in the jeju swimbladder.

While the connection between hyperoxia and the generation of ROS has been unequivocal, the relation between hypoxic conditions and the formation of ROS has been debated intensively. It was originally expected that the reduction in oxygen availability would result in a concomitant decrease in ROS production because oxygen is required for the generation of ROS (Welker et al. 2013). Meanwhile, several studies have shown, however, that reduced oxygen availability (hypoxia) may also cause oxidative stress (Welker et al. 2013). In fish, hypoxia-induced oxidative stress has been detected in several species, including goldfish *Carassius auratus* (Lushchak et al. 2001), common carp *Cyprinus carpio* (Lushchak et al. 2005b), rotan *Perccottus glenii* (Lushchak and Bagnyukova 2007), medaka (*Oryzias latipes*) (Lushchak and Bagnyukova 2007; Oehlers et al. 2007), piapara (*Leporinus elongatus*) (Wilhelm Filho et al. 2005) and the Indian catfish *Clarias batrachus* (Tripathi et al. 2013).

To prevent tissue damage in situations of variable oxygen availability when there is the danger of inordinate accumulation of ROS, animals have developed a defense system for rapidly breaking down ROS (Storey 1996; Wilhelm Filho 1996). This system consists of low molecular weight antioxidants such as glutathione (GSH + GSSG), thioredoxin (Trx-(SH_2_)/Trx-SS-Trx), ascorbic acid (vitamin C), retinol (vitamin A) or α-tocopherol (vitamin E). In addition, several enzymes can remove ROS, such as catalase and superoxide dismutase (SOD). SOD catalyzes the dismutation of O_2_^−^ into O_2_ and H_2_O_2_, and catalase decomposes H_2_O_2_. Glutathione peroxidase (GPx) also decomposes H_2_O_2_ using the GSH in its reduced form, and glutathione reductase (GR) subsequently reduces GSSG back to GSH at the expense of NADPH_2_ (Hermes-Lima 2004). Oxidized NADP^+^ may then be reduced again by the activity of glucose-6-phosphate dehydrogenase and 6-phosphogluconate dehydrogenase, enzymes of the pentose phosphate shunt.

By exposing fish to variable oxygen conditions (hypoxia as well as hyperoxia), it has been shown that the ROS defense system is quite flexible. Hyperoxic exposure not only induces an increased activity of ROS-degrading enzymes (Lushchak et al. 2005a; Lushchak and Bagnyukova 2006; Lushchak 2011) but also hypoxia often results in an increase in the activity of these enzymes (Lushchak et al. 2001, 2005b; Wilhelm Filho et al. 2005; Lushchak and Bagnyukova 2007; Tripathi et al. 2013). The latter may be the response to an increase in ROS under hypoxic conditions, but it may also be a preparation of the organism for oxidative stress encountered during recovery from hypoxia, and the concept of a preparation for oxidative stress is widely accepted (Welker et al. 2013). In the present study, we, therefore, hypothesized that the ROS defense system of the two erythrinids, jeju (*Hoplerythrinus unitaeniatus*) and traira (*Hoplias malabaricus*), would also show this flexibility to changing oxygen availability. We also hypothesized that the swimbladder of the air-breathing jeju, depending on the air-breathing activity, is frequently facing changing oxygen partial pressures, while the swimbladder of the non-air-breathing traira was expected to face more or less constant low-oxygen partial pressures. Based on the results obtained, we intended to assess the possible contribution of the swimbladder to gas exchange.

## Materials and methods

Experiments were performed at the Instituto Nacional de Pesquisas da Amazônia (INPA). All procedures were in compliance with Brazilian national and INPA animal care regulations.

Erythrinid fish used for this study, the jeju and the traira, were caught by INPA fishermen, brought to the INPA and kept in outdoor fish tanks, supplied with running INPA freshwater. The water was continuously aerated. In the tanks, the fish had free access to air and could, therefore, breathe air voluntarily. Fish were fed daily until the day before the experiment. Water pH was 6.0 ± 0.05, the temperature was 26 ± 1 °C.

### Surgical procedure in jeju

Jeju were anaesthetized with neutralized MS222 (0.1 g L^− 1^). The body wall was opened close to the lateral line below the end of the dorsal fin. A Clay-Adams PE50 catheter (Becton, Dickinson and Co., Franklin Lakes, NJ, USA), slightly flared at the insertion end, was inserted into the caudal end of the posterior swimbladder and anchored in place with a surgical suture and a drop of cyanoacrylate tissue glue (Vetbond™, 3M Co., Maplewood, MN, USA). The catheter was guided through the muscle tissue and the skin using a PE160 sleeve, which was anchored to the PE50 with the glue, then sutured to the body surface. After surgery, fish were transferred to 25-L plastic tanks with aerated INPA freshwater for a recovery period of at least 16 h.

### Behavioral observations and in vivo measurements of swimbladder oxygen in jeju

Jeju with a catheter inserted into the posterior swimbladder were transferred to translucent 2-L plastic tanks, filled with INPA freshwater until about 6 cm below the top of the tank so the fish could breathe air freely. Outside the tank, the catheter was connected to a gas-tight glass syringe so that gas samples from the posterior swimbladder could be taken without disturbing the fish.

Water was aerated using air stones (normoxia = 7–8 mg O_2_ L^− 1^, corresponding to a PO_2_ of 20 kPa). After an acclimation period of at least 2 h, fish were observed for 30 min by counting every air-breath. Air-breaths could easily be identified because immediately after the fish left the surface, exhaled air bubbles left the opercular cavity via the operculum. Then the water PO_2_ was lowered to 1–1.5 mg O_2_ L^− 1^ (corresponding to a PO_2_ of 2.6–3.9 kPa or about 17% saturation; hypoxia) by bubbling nitrogen into the tank. After the water was equilibrated to the new PO_2_, air-breathing activity was again monitored for 30 min by counting every air breath. At the end of this hypoxia treatment, water was aerated and the fish were allowed a recovery period of 2 h. Following this recovery period, water was bubbled with pure oxygen to achieve hyperoxic conditions (28–32 mg O_2_ L^− 1^, corresponding to a PO_2_ of 73–83 kPa or about 450% saturation). Air-breathing activity was again counted for 30 min. Water oxygen levels were recorded with a portable oxygen electrode and meter (WTW Multi3410 meter, Weilheim, Germany) for normoxic and hypoxic conditions. Hyperoxic conditions were verified using a Clark-type oxygen electrode and the appropriate meter (Radiometer, E5047 electrode, connected to a PHM 73 m, Copenhagen, Denmark).

Swimbladder gas samples of 0.2–0.3 mL were taken at the end of the control period, and at the end of the hypoxic or hyperoxic incubation. After disconnecting the syringe from the catheter, the syringe was immediately sealed with parafilm and the PO_2_ of the gas sample was measured using a PreSens optical oxygen probe (PreSens Precision Sensing, Regensburg, Germany), inserted into a 22G needle. With this needle, the parafilm blocking the tip of the glass syringe containing the gas sample was penetrated and the oxygen probe advanced to measure the PO_2_ of the gas sample. After the measurement, the gas sample was returned to the posterior swimbladder. The probe was calibrated with air and nitrogen in the gas phase. Aerial oxygen was set to 100% (20 kPa), and nitrogen was set to zero oxygen.

### Hypoxic and hyperoxic incubations in jeju and traira

Fish were transferred to individual 30-L plastic tanks the day before the experiment. Water was aerated using air stones. The next morning, 75% of the water was siphoned out of the tank and replaced with fresh water. For hypoxic conditions, water oxygen was lowered to 1–1.5 mg O_2_ L^− 1^ (corresponding to a PO_2_ of 2.6–3.9 kPa; hypoxia) by bubbling nitrogen into the tank. Fish were incubated under hypoxic conditions for 4 h. Hyperoxic conditions (28–32 mg O_2_ L^− 1^, corresponding to a PO_2_ of 73–83 kPa; incubation time 4 h) were achieved by bubbling oxygen. Control fish were incubated in aerated normoxic water for 4 h. Water oxygen partial pressure was monitored using the WTW portable oxygen electrode for hypoxic and normoxic conditions, and the Radiometer Clark-type oxygen electrode for hyperoxic conditions, as above.

### Tissue preparation in jeju and traira

Fish maintained for 4 h under normoxic, hypoxic or hyperoxic conditions were rapidly anesthetized with an overdose of neutralized tricaine methanesulfonate **(**MS222; 0.5 g L^− 1^) and killed by a sharp blow on the head. To prevent further blood flow to the swimbladder and possible modification of swimbladder gas, fish were then immediately injected with 1 mL of 100 mM KCl into the caudal vein. Preliminary tests showed that this stopped the heart within 30 s. Fish were opened laterally and a small portion of the swimbladder was carefully exposed for measurement of PO_2_ of the swimbladder using a PreSens oxygen probe housed in a 22G needle, as above. After penetrating the posterior section of the swimbladder, the probe was carefully advanced into the swimbladder lumen and the PO_2_ recorded. Subsequently, the oxygen tension of the anterior section of the swimbladder was recorded, using a separate penetration. The whole procedure starting with anesthesia and recording the oxygen tension of the posterior bladder took between 2 and 3 min, and within less than 4 min recording of swimbladder oxygen tensions was completed.

After recording gas tensions, the swimbladder was quickly dissected. Connective tissue was removed and the remaining tissue was carefully rinsed with saline, cleaned and blotted dry. The anterior swimbladder tissue of jeju and traira and also the posterior part of traira swimbladder was dissected into small portions and immediately frozen in liquid nitrogen. For jeju, only the vascularized (anterior) section of the posterior swimbladder tissue was used for tissue preparation. Tissues were then stored in a biofreezer at − 80 °C until further analysis.

### Biochemical analyses

For determination of total glutathione (GSSG + GSH) content of the frozen tissue samples, tissue extracts were prepared using 5% metaphosphoric acid (MPA). The frozen tissues were ground to a fine powder and dissolved 1:5 w/v in 5% MPA. Under ice cooling, the solution was homogenized using a motorized homogenizer. Extracts were centrifuged at 13,000 rpm for 15 min at 4 °C and the supernatant was diluted using assay buffer of the GSSG + GSH Assay kit (STA-312; Cell Biolabs, Inc San Diego, USA). GSSG + GSH concentration was determined using the OxiSelect Total Glutathione (GSSG + GSH) Assay Kit (STA-312) Cell Biolabs, Inc San Diego, USA, following the manufacturer’s instructions.

For measurement of enzyme activities, the frozen tissue samples were homogenized on ice in 1:5 w/v of ice-cold homogenization buffer (10 mM of TRIS/HCl, 0.1 mM of disodium EDTA, 150 mM of NaCl, pH 7.5 at 25 °C). Under ice cooling, the tissue was homogenized using a motorized homogenizer. Homogenates were centrifuged at 13,000 rpm for 15 min at 4 °C and appropriate dilutions of the supernatant were used for the enzyme and protein assays.

Enzyme activities were measured using a SpectraMax 384Plus microplate spectrophotometer (Molecular Devices, Sunnyvale, CA, USA) with temperature control at 25 ± 0.1 °C. Glutathione reductase (GR; EC 1.6.4.2.) and glutathione peroxidase (GPx; EC 1.11.1.9.) activities were measured using the Glutathione Reductase Assay Kit (No 703202; Cayman Chemical Company, Ann Harbor, USA), and the Glutathione Peroxidase Assay Kit (No 703102; Cayman Chemical Company, Ann Harbor, USA).

Catalase (Cat; EC 1.11.1.6.) activity was assayed using the Amplex Red Catalase Assay Kit (A22180; Molecular Probes, Eugene, USA). Superoxide dismutase (SOD; EC 1.15.1.1.) activity was measured following a procedure described by McCord and Fridovich (McCord and Fridovich 1969). Briefly, reactive oxygen species generated from xanthine in the xanthine oxidase reaction cause a reduction of cytochrome c, which is inhibited by the presence of SOD. One unit of SOD activity is defined as the amount of enzyme (per milligram of protein) that inhibits the reduction of cytochrome c observed in the blank without SOD by 50%.

Protein concentration in the homogenate was measured with Coomassie Brilliant Blue G-250 (Bradford 1976) using bovine serum albumin as a standard.

### Statistics

Data are expressed as mean ± 1 SEM with N giving the number of animals analyzed in each species. GSSG + GSH concentrations are given as nmol g^−1^wwt (wet weight), enzyme activities as U mg^−1^protein (µmol min^− 1^ mg^− 1^protein) or as mU mg^− 1^protein (nmol min^− 1^ mg^− 1^protein). For statistical analysis three-way repeated measures ANOVA, followed by the Holm-Sidak Multiple Comparison procedures, was used. Fish species (jeju, traira), tissue (anterior bladder, posterior bladder) and incubation PO_2_ (normoxia, hypoxia, hyperoxia) were used as parameters (factors) 1, 2 and 3, and enzyme activity or GSH + GSSG concentration as variables (data). PO_2_ values of the anterior and posterior swimbladder of jeju and traira were analyzed using three-way ANOVA followed by the Bonferoni multiple comparison procedures. PO_2_ values of the anterior and posterior swimbladder of catheterized jeju were analyzed using one-way ANOVA followed by the Holm-Sidak multiple comparison test. In rare cases, the normality test or equal variance test failed using the original data. In this case the data were log transformed prior to statistical analysis. The statistical analysis was performed using SigmaPlot 12.0. Trend/regression lines describing the relation between PO_2_ and number of air breaths were calculated using Excel using a linear and a logarithmic fit. Significant differences between values were accepted for *p* < 0.05.

## Results

The mean body mass of jeju used for the experiments was 95.6 ± 5.9 g (mean ± SEM), and the average fork length was 19.6 ± 0.4 cm (*N* = 26). The mean body mass of traira amounted to 344.1 ± 21.9 g (mean ± SEM), and the average fork length was 31.4 ± 0.7 cm (*N* = 22). For both species, body mass and fork length of the individual experimental groups were tested separately and there was no difference in body mass or fork length between control, hypoxic or hyperoxic animals.

**Fig. 1:**
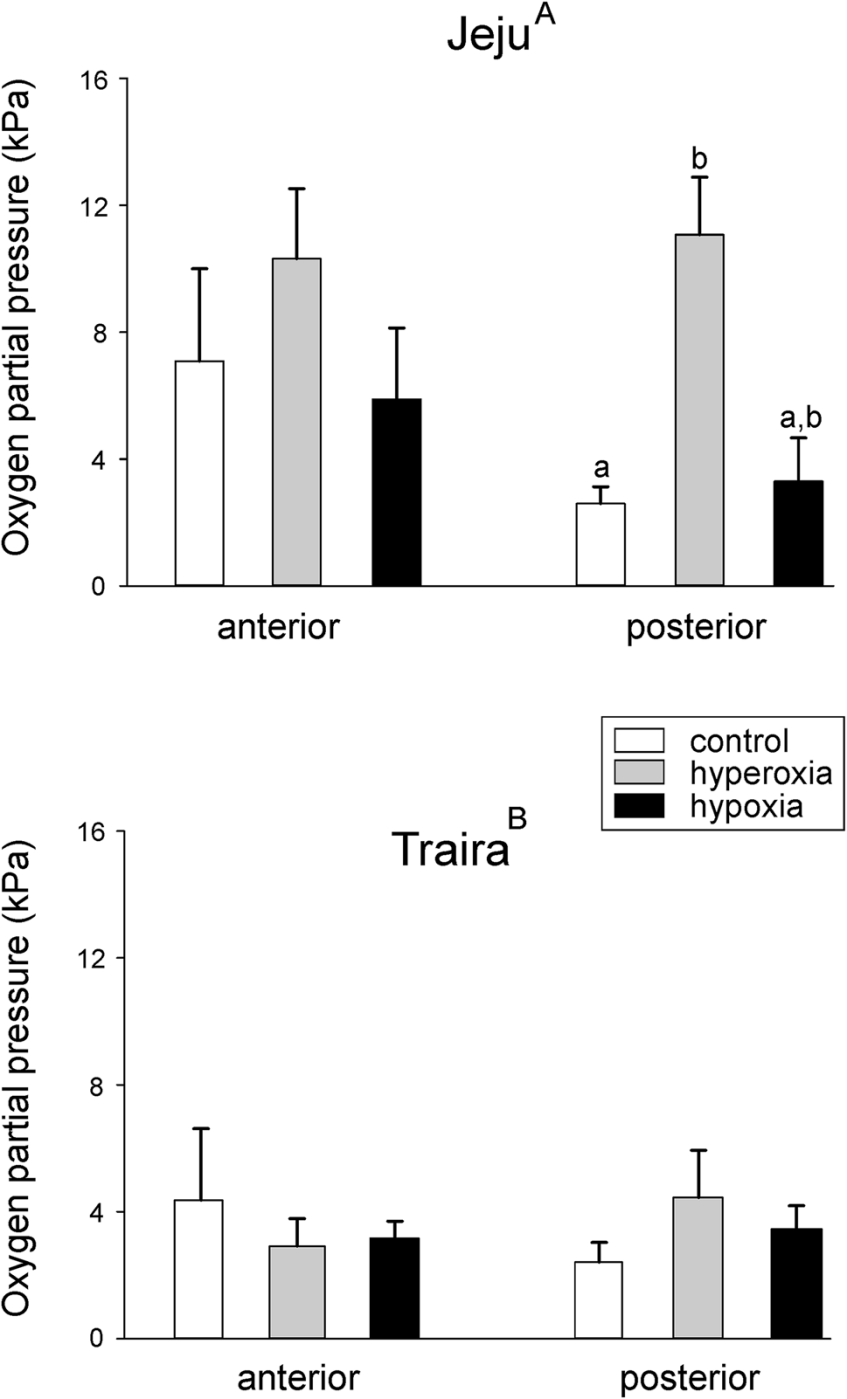
Oxygen content in kPA measured by direct puncture in the anterior and posterior swimbladder of jeju (**a**) and traira (**b**) under normoxic, hypoxic and hyperoxic conditions. Different capital letters denote significant differences between species, small letters denote significant differences within a species (*p* < 0.05), bars without letters are not significantly different; *N* is given in brackets below the columns. There was a significant overall difference between the two species

Under normoxia, direct measurement of PO_2_ in the swimbladder on terminally sampled jeju, by direct penetration of the bladder with the oxygen probe, revealed a value of 7.08 ± 2.9 kPa in the anterior bladder, and only 2.6 ± 0.54 kPa (*N* = 6) in the posterior bladder (Fig. 1a). In traira under normoxia, a PO_2_ of 4.36 ± 2.24 kPa was detected in the anterior bladder, and 2.42 ± 0.62 kPa (*N* = 6) in the posterior bladder by the same technique (Fig. 1b). For both species, there was no significant difference between the two parts of the swimbladder.

In jeju, while 4 h of hypoxic incubation did not cause any change in anterior and posterior swimbladder PO_2_, 4 h of hyperoxia resulted in a significantly elevated PO_2_ in the posterior bladder with 11.08 ± 1.8 kPa (*N* = 5; Fig. 1a). PO_2_ in the anterior bladder was also elevated to approximately the same value, but the difference was not yet significant.

Visual observation of the swimming activity of traira revealed almost no surfacing activity under any of the experimental treatments. Fish were quiet on the bottom of the tank, and even ventilatory activity was hardly detectable under hypoxic conditions.

In traira neither 4 h of hypoxia (1–1.5 mg O_2_ L^− 1^) nor 4 h of hyperoxia (28–32 mg O_2_ L^− 1^) caused any significant change in the PO_2_ of the anterior and posterior swimbladder (Fig. 1b). A comparison of swimbladder PO_2_ between the two species revealed significantly higher levels of oxygen in jeju under both hyperoxic and hypoxic conditions (Fig. 1).

**Fig. 2:**
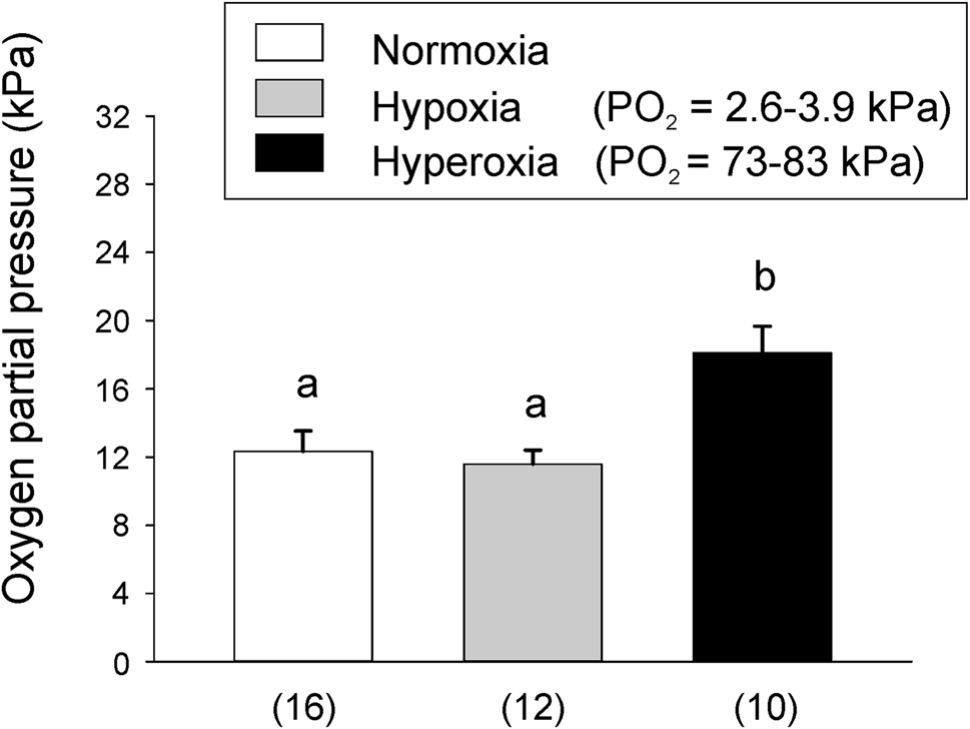
Oxygen content in kPa measured via a catheter in the posterior swimbladder of jeju under normoxic, hypoxic and hyperoxic conditions. Different small letters denote significant differences between treatments (*p* < 0.05); *N* is given in brackets below the columns

Oxygen partial pressure in the posterior bladder of jeju measured on live, freely swimming animals via a catheter chronically inserted at the caudal end of the bladder revealed significantly higher PO_2_ values (Fig. 2) as compared to the values obtained by puncturing the swimbladder wall and using the optical fiber (Fig. 1a). Under control conditions, the oxygen partial pressure amounted to 12.34 ± 1.2 kPa (*N* = 16). A similar value was recorded after 30 min of hypoxia, but after 30 min of hyperoxia PO_2_ was significantly elevated to 18.1 ± 1.56 kPa (*N* = 10; Fig. 2). This is similar to the trend seen in the terminally sampled jeju after 4 h of incubation under hyperoxia (Fig. 1a), where the PO_2_ was also elevated as compared to normoxia. As also observed in the animals incubated for 4 h under hypoxic or hyperoxic conditions, hypoxia stimulated air-breathing activity, but under hyperoxic conditions no air-breathing activity was observed.

**Fig. 3:**
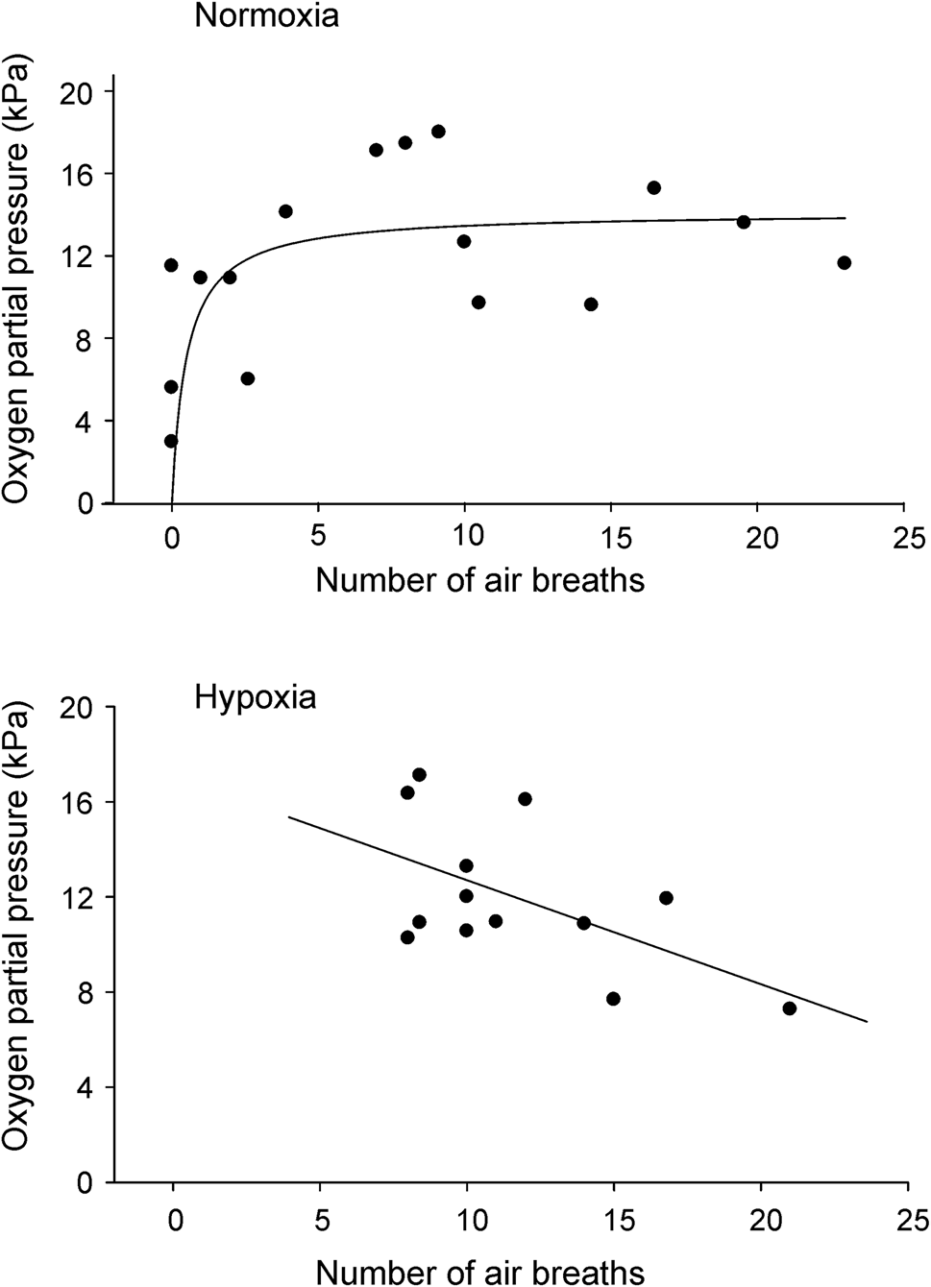
Oxygen content in kPa measured via a catheter in the posterior swimbladder of jeju in relation to the number of breaths taken within an observation period of 30 min in **a** normoxic and **b** hypoxic jeju. Under hyperoxic conditions, no air-breathing activity was observed

In Fig. 3a, the oxygen partial pressure of the posterior swimbladder of jeju under normoxia, as sampled by catheter in freely swimming animals, was plotted against the number of air-breaths taken in the preceding 30 min. The data showed a remarkable variability, but the overall relationship appeared to be hyperbolic and was well described by the equation:

1 $$f\,=\,0.{\text{5699ln}}\left( x \right)\,+\,{\text{11}}.{\text{764}}\;\;\left( {{R^{\text{2}}}\,=\,0.{\text{3642}};\;\;{\text{ }}f\,=\,{\text{P}}{{\text{O}}_{\text{2}}}\;\;{\text{of the bladder}};{\text{ }}x\,=\,{\text{number of breaths taken}}} \right).$$

Thus, PO_2_ initially increased with increasing air-breathing activity and the lowest PO_2_ was detected in fish that did not breathe air during the observation period. Between 5 and 25 breaths per 30 min, however, almost no further increase in the PO_2_ was detectable.

Under hypoxic conditions, the scatter of the data was reduced, and the PO_2_ of the bladder decreased in a linear fashion with increasing number of breaths taken (Fig. 3b), as described by the equation:

2 $$f= - \;0.{\text{4374}}x\,+\,{\text{17}}.0{\text{72}}\;\;\left( {{R^{\text{2}}}\,=\,0.{\text{3173}};\;\;{\text{ }}f\,=\,{\text{P}}{{\text{O}}_{\text{2}}}\;{\text{of the bladder}};{\text{ }}x\,=\,{\text{number of breaths taken}}} \right).$$

The number of breaths taken under hypoxic conditions (11.7 ± 1.1 breaths/30 min; *N* = 13) was significantly higher than under normoxic conditions (7.9 ± 1.8 breaths/30 min; *N* = 16).

**Fig. 4:**
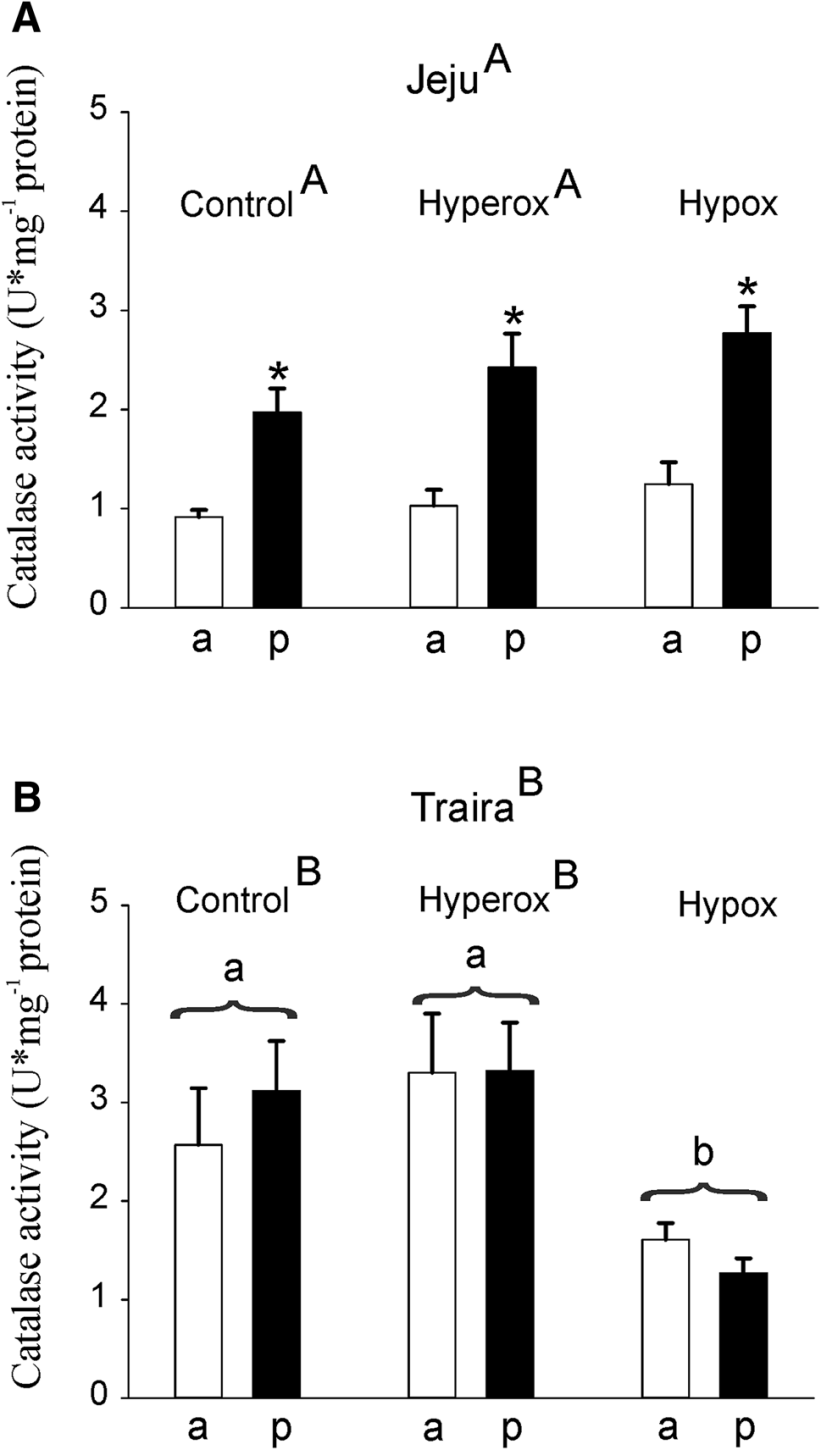
Catalase activity in U mg^− 1^ protein in anterior (a) and posterior (p) swimbladder tissue of jeju (**a**) and traira (**b**) under normoxic, hyperoxic and hypoxic conditions. Asterisks mark significant differences (*p* < 0.05) between posterior and anterior values within the same species and treatment. Capital letters denote significant differences between the two species, different small letters denote significant differences between treatments within a species, bars without letters are not significantly different. There was also a significant overall difference between the two species. Jeju: control, *N* = 6, hyperoxia, *N* = 9, hypoxia, *N* = 8; traira: control, *N* = 15, hyperoxia, N = 9, hypoxia, *N* = 9

As a next step, we measured the activity of ROS-degrading enzymes in anterior and posterior swimbladder tissue of both species following incubation under normoxic control conditions, after 4 h of hypoxia and after 4 h of hyperoxia. In jeju, catalase activity in the posterior section of the swimbladder was significantly higher than in the anterior section under all three conditions tested, but there was no significant difference between normoxic, hypoxic- or hyperoxic-incubated fish (Fig. 4a). In traira, no difference was detected between the anterior and the posterior part of the swimbladder, but following 4 h of hypoxia-catalase activity was significantly lower by about 50% in both parts of the swimbladder as compared to normoxic- or hyperoxic-incubated fish (Fig. 4b). The overall comparison revealed significant differences between the two species, with specific significant differences in normoxia and hyperoxia, but not in hypoxia. Catalase activity in the posterior section of traira was significantly different from the activity measured in the posterior section of jeju.

**Fig. 5:**
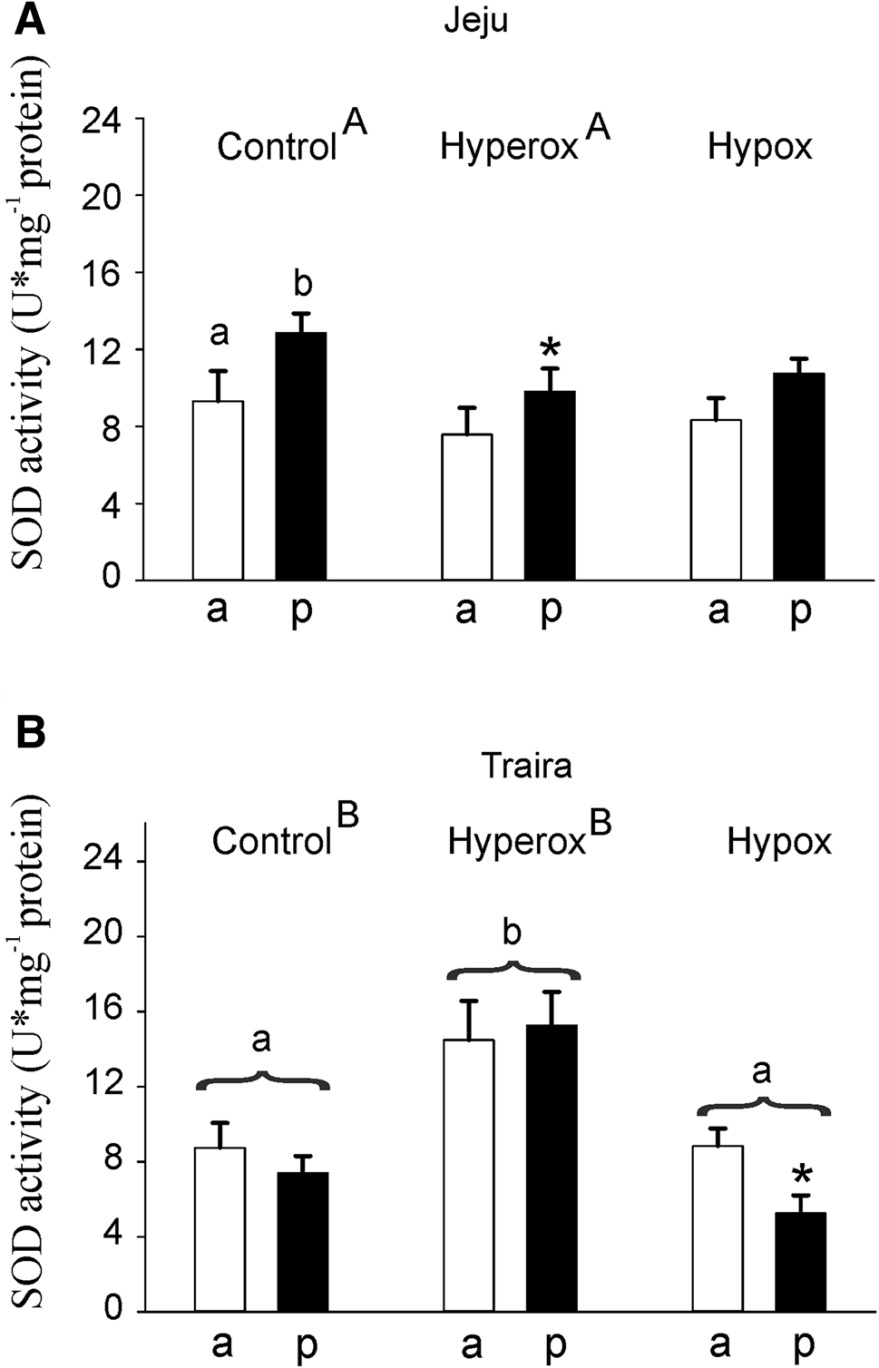
Superoxide dismutase (SOD) activity in U mg^− 1^ protein in anterior (a) and posterior (p) swimbladder tissue of jeju (**a**) and traira (**b**) under normoxic, hyperoxic and hypoxic conditions. Asterisks mark significant differences (*p* < 0.05) between posterior and anterior values within the same species and treatment. Capital letters denote significant differences between the two species, different small letters denote significant differences between treatments within a species, bars without letters are not significantly different. Jeju: control, *N* = 11, hyperoxia, *N* = 8, hypoxia, *N* = 8; traira: control, *N* = 14, hyperoxia, *N* = 9, hypoxia, *N* = 9

Superoxide dismutase activity measured in jeju swimbladder tissue was similar in both sections of the swimbladder under normoxic and hypoxic conditions, but under hyperoxia SOD activity in the posterior bladder was higher than in the anterior bladder (Fig. 5a). In jeju, overall no differences could be detected in response to hypoxic or hyperoxic incubation (Fig. 5a). In traira, however, hyperoxic conditions caused a 1.6- to two-fold increase in SOD activity in the anterior and posterior swimbladder, respectively, and this was significantly different from control values as well as from hypoxic values (Fig. 5b). Under hypoxic conditions, SOD activity in the posterior bladder was significantly lower than in the anterior bladder. The comparison between the two species showed that under normoxic control conditions, SOD activity in jeju was significantly higher than in traira. However, under hyperoxic conditions, SOD activity was significantly higher in traira, as compared to jeju.

**Fig. 6:**
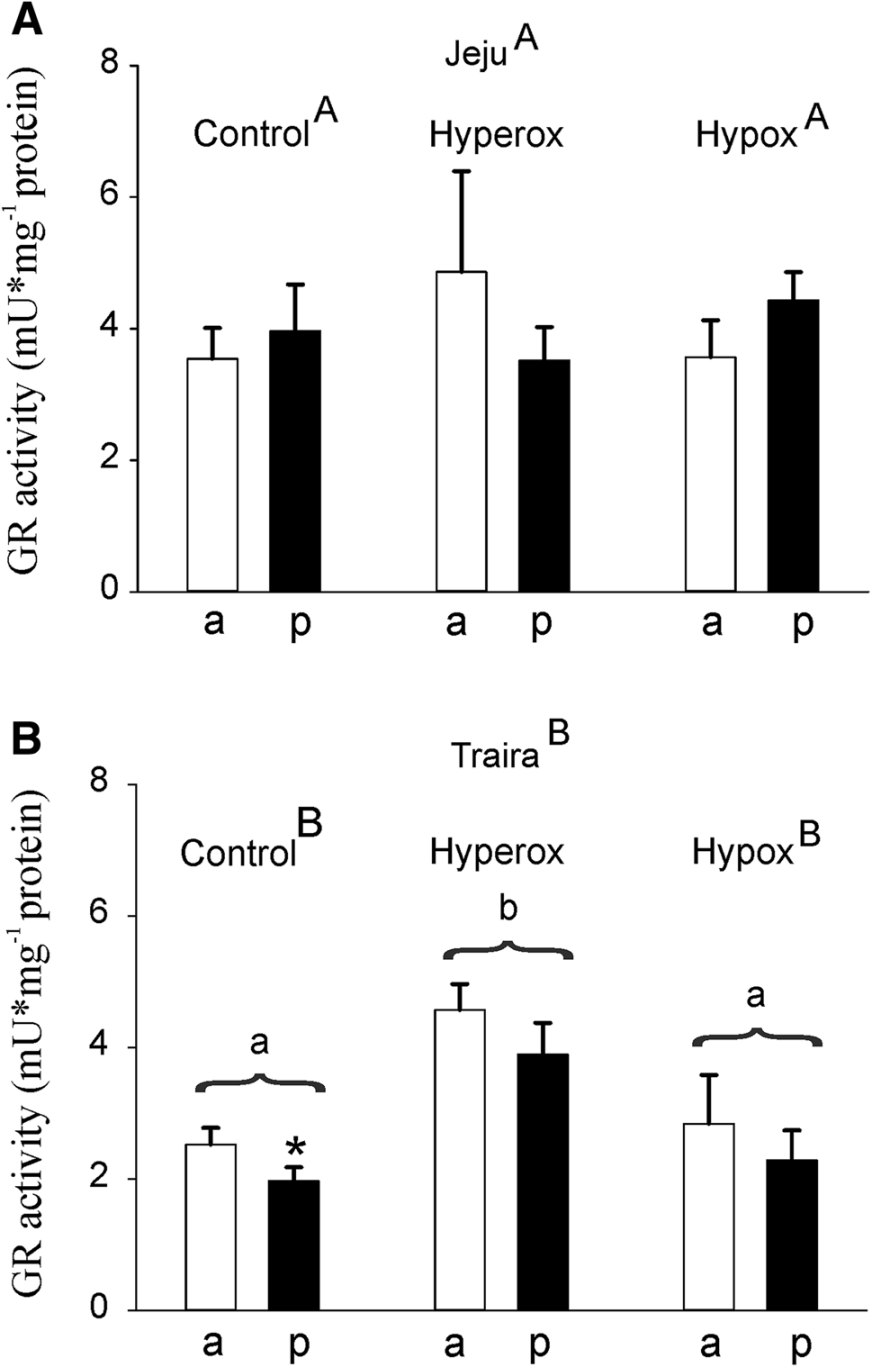
Glutathione reductase (GR) activity in mU mg^− 1^ protein in anterior (a) and posterior (p) swimbladder tissue of jeju (**a**) and traira (**b**) under normoxic, hyperoxic and hypoxic conditions. Asterisks mark significant differences (*p* < 0.05) between posterior and anterior values within the same species and treatment. Capital letters denote significant differences between the two species, different small letters denote significant differences between treatments within a species, bars without letters are not significantly different. There was also a significant overall difference between the two species. Jeju: control, *N* = 6, hyperoxia, *N* = 8, hypoxia, *N* = 8; traira: control, *N* = 14, hyperoxia, *N* = 9, hypoxia, *N* = 8

Measurement of GR activity in jeju swimbladder revealed similar activity in both sections of the swimbladder, and no differences could be detected in response to hypoxic or hyperoxic incubation (Fig. 6a). In traira anterior and posterior swimbladder tissue, GR activity was also quite similar, but again, following hyperoxic incubation, the activity in both sections of traira swimbladder was significantly higher than under normoxia or following hypoxic incubation (Fig. 6b). In hyperoxia, GR activity was as almost twice as high as in normoxia. The comparison of jeju and traira showed overall significant difference between the two species. Specifically, there was significantly higher GR activity in jeju swimbladder tissue under normoxic conditions and under hypoxia, but no difference was detected under hyperoxic conditions.

**Fig. 7:**
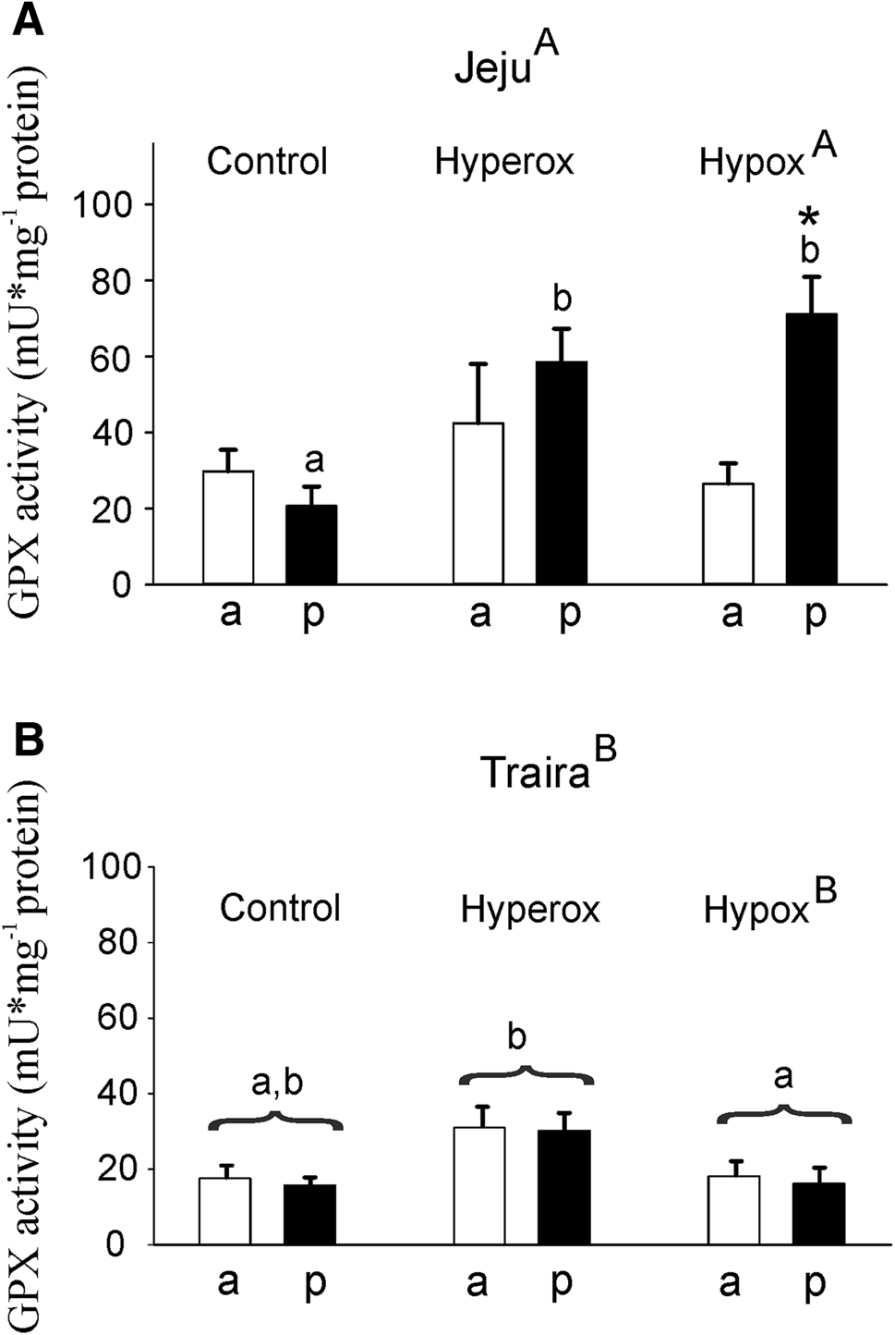
Glutathione peroxidase activity in mU mg^− 1^ protein in anterior (a) and posterior (p) swimbladder tissue of jeju (**a**) and traira (**b**) under normoxic, hyperoxic and hypoxic conditions. Note the, different scales in panels **a** and **b**. Asterisks mark significant differences (*p* < 0.05) between posterior and anterior values within the same species and treatment. Capital letters denote significant differences between the two species, different small letters denote significant differences between treatments within a species, bars without letters are not significantly different. There was also a significant overall difference between the two species. Jeju: control, *N* = 10, hyperoxia, *N* = 9, hypoxia, *N* = 5; traira: control, *N* = 11, hyperoxia, *N* = 9, hypoxia, *N* = 6

Under normoxia, GPx activity was similar in the anterior and posterior section of jeju swimbladder (Fig. 7a). Hypoxic as well as hyperoxic incubation resulted in a significant enhancement of GPx activity in the posterior part of jeju swimbladder. Glutathione peroxidase activity increased 2.8-fold under hyperoxic conditions and 3.4-fold under hypoxic conditions. In traira, GPx activities in the anterior and posterior section of the swimbladder were always similar to each other, but varied according to the treatment (Fig. 7b). Under hyperoxia, GPx activity was significantly elevated as compared to hypoxia, but it was not different from the activity measured under control conditions. Overall, GPx activity was significantly higher in jeju as compared to traira, though a specific significant difference was seen only in the hypoxia treatment.

**Fig. 8:**
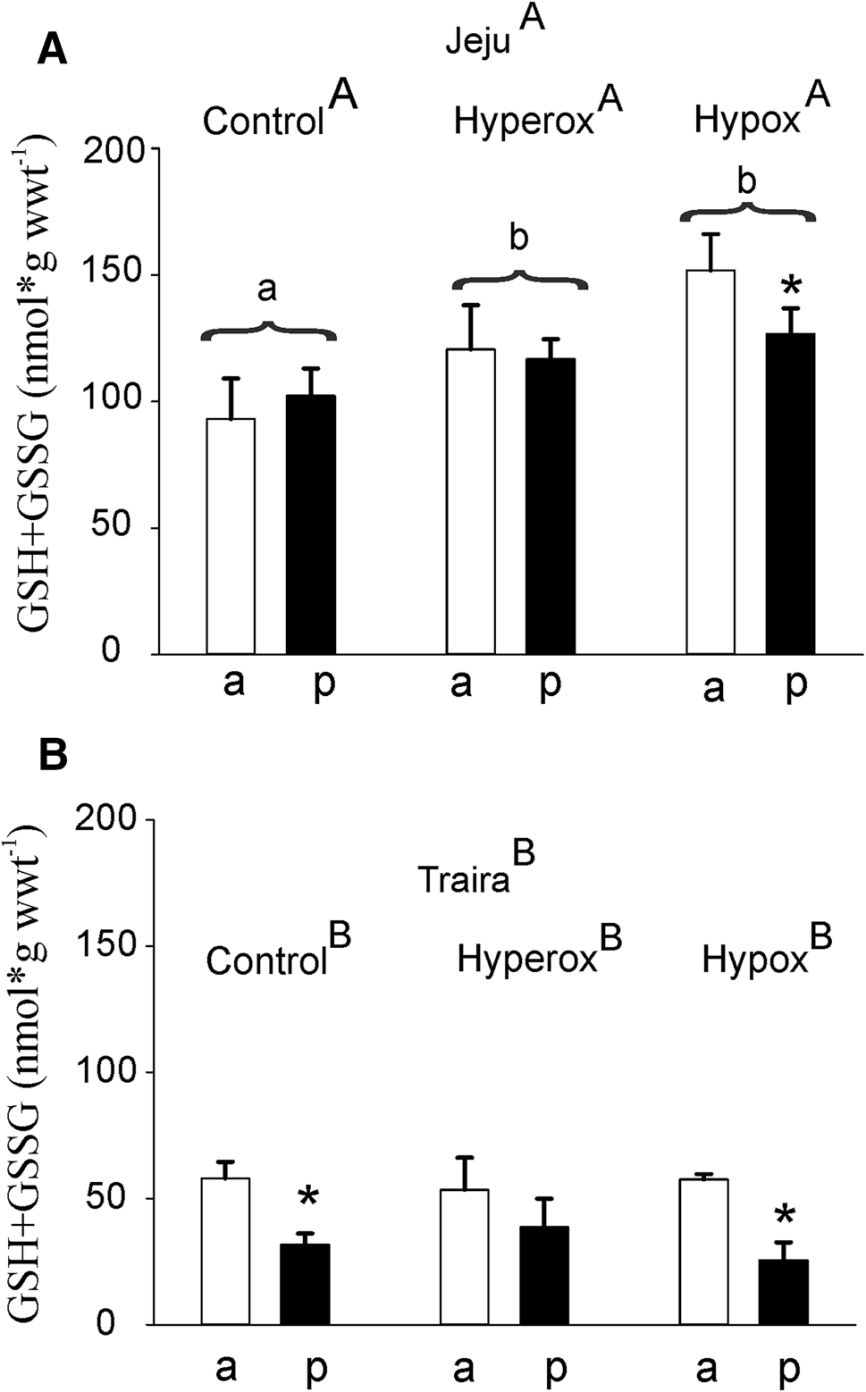
Total GSSG + GSH concentration in anterior (a) and posterior (p) swimbladder tissue of jeju (**a**) and traira (**b**) under normoxic, hyperoxic and hypoxic conditions. Asterisks mark significant differences (*p* < 0.05) between posterior and anterior values within the same species and treatment. Capital letters denote significant differences between the two species, different small letters denote significant differences between treatments within a species, bars without letters are not significantly different. There was also a significant overall difference between the two species. Jeju: control, *N* = 6, hyperoxia, *N* = 7, hypoxia, *N* = 8; traira: control, *N* = 6, hyperoxia, *N* = 3, hypoxia, *N* = 6

Measurement of total glutathione concentration (GSH + GSSG) again resulted in similar concentrations in the anterior and posterior section of jeju swimbladder. Comparing the values obtained under normoxic, hyperoxic and hypoxic conditions, however, showed a significant elevation of total glutathione concentration under hypoxic as well as under hyperoxic conditions (Fig. 8a). In traira, similar glutathione concentrations were detected under all conditions in both sections of the swimbladder. In the anterior section, the concentration always appeared to be higher, and this difference was significant for control and for hypoxic fish. Comparing the jeju with traira revealed significantly higher concentrations of total glutathione in jeju under all conditions.

## Discussion

### Critique of methods

Swimbladder puncture with a 22G needle and a fiber optic system has been used to measure oxygen content in the anterior and posterior swimbladder in quickly sacrificed animals, and chronic catheterization by inserting PE50 tubing into the caudal end of the posterior bladder in freely swimming animals. The results obtained with the catheter were significantly higher and consistent with values reported by Kramer (Kramer 1978), who collected gas by puncturing the posterior tip of the bladder and collecting gas in a water-filled funnel, and by Farrell and Randall (Farrell and Randall 1978), who cannulated the posterior bladder similar to our procedure. Comparing the changes of jeju posterior bladder oxygen content under normoxic, hypoxic and hyperoxic conditions, both procedures gave similar results qualitatively. Incubation under hypoxic conditions did not change posterior bladder oxygen content, but under hyperoxia, oxygen content increased. Both procedures appeared to have intrinsic limitations. Using the direct puncture method, the fish had to be anesthetized, and the swimbladder had to be exposed by dissection before the swimbladder wall could be penetrated with the 22G needle. Although the whole procedure was completed within 2 or 3 min, oxygen could have been removed from the bladder during this time and not replaced, resulting in lower oxygen values. In a previous study, we measured an oxygen uptake of 5.6 ± 0.6 µmol g^− 1^ h^− 1^ for jeju at a temperature of 29–32 °C (Wood et al. 2016). Therefore, a 100 g of jeju would consume about 19 µmol of oxygen within 2 min. A 100 g of jeju would have a swimbladder volume of about 5 mL (Kramer 1978). Assuming an oxygen content of 60% (12 kPa) of aerial oxygen (measured in the catheter experiments), this would give an oxygen content of about 0.6 mL, equivalent to 26 µmol at STPD. Accordingly, given the time needed for anaesthesia and the further up to 30 s for the time needed for the injection of KCl to stop the heart (1.5–2 min total), the continuation of blood flow could indeed have appreciably reduced the oxygen content of the posterior swimbladder.

A problem of the catheter in turn is the tightness, and a tiny leak in the gas phase or diffusion through the PE tubing may have caused an increase in the oxygen content. The swimbladder typically contains some moisture to avoid desiccation of the epithelia, which sometimes hinders the collection of a clean gas sample. The dead space of the catheter may be equilibrated with a mixture of atmospheric and swimbladder gas, which also may result in elevated gas contents. Thus, while the direct penetration method may result in an underestimation of swimbladder oxygen content, the catheter may result in an overestimation. We, therefore, assume the real value to be in between these two results. Convincingly, however, both measurements consistently showed that jeju-posterior swimbladder oxygen content was not modified under hypoxic conditions, but it significantly increased under hyperoxic conditions.

### Swimbladder function under hypoxia and hyperoxia

As expected, the swimbladder of the jeju had a significantly higher oxygen content than the swimbladder of traira. Gases are resorbed from non-ventilated cavities according to their physical solubility, resulting in an accumulation of the less soluble gases (Piiper et al. 1962; Piiper 1965). The swimbladder of traira is used as a buoyancy structure and hardly ventilated (Rantin et al. 1992; Val and Almeida-Val 1995; Glass and Rantin 2009; Pelster et al. 2016), therefore, the less soluble nitrogen should accumulate in this bladder. The jeju swimbladder, in turn, is frequently ventilated, and the inhaled gas initially enters the anterior section of the swimbladder (Randall et al. 1981). The posterior part of the jeju swimbladder is filled from the anterior part. It is highly vascularized and used as a gas exchange organ, and we measured a lower PO_2_ in posterior bladder as compared to the anterior part, although this difference was not yet significant.

Under normoxic conditions a large scatter in the oxygen content of the swimbladder was observed, which in part was attributable to the time difference between the last breath and sampling of swimbladder gas. In line with the results obtained for traira, the lowest oxygen content in jeju under normoxia was detected in swimbladders that were hardly ventilated. Between 5 and 25 breaths per 30 min, however, oxygen content was quite variable and no clear correlation with the number of breaths taken was detectable. In addition to ventilation the intensity with which the bladder was used as an oxygen resource must have an influence, and removal of oxygen from the bladder is dependent on blood flow. In an intensely ventilated swimbladder with frequent gas exchange between the anterior and the posterior bladder, the differences in oxygen content between the two parts is expected to become smaller.

Under hypoxic conditions, ventilatory activity of the jeju increased, as reported also in previous studies (Kramer 1978; Stevens and Holeton 1978; Oliveira et al. 2004; Perry et al. 2004; Juca-Chagas 2004; Pelster et al. 2016), but the oxygen content did not increase. Indeed, a negative correlation between posterior bladder oxygen content and breathing frequency was observed. Testing the PO_2_ in the gas space above the hypoxic water revealed that in this set up, PO_2_ in the gas space may drop to 15–20% below aerial PO_2_ (i.e. to 16–17 kPa). Thus, air-breathing jeju would pick up slightly hypoxic air, but nevertheless the PO_2_ of this air was much higher than water PO_2_ with a value of only 2.6–3.9 kPa. The constant PO_2_ in the swimbladder, therefore, suggests that the intensely ventilated swimbladder is indeed used to supply oxygen to other tissues.

In traira under hyperoxic conditions, swimbladder oxygen content was not different from control or hypoxia-incubated animals, supporting the notion that the swimbladder is not used for gas exchange and not intensely perfused. Accordingly, changes in arterial PO_2_ caused by changing water PO_2_ will only in the long run influence swimbladder PO_2_. In the jeju, however, oxygen content of the posterior bladder increased significantly, as observed in both directly punctured swimbladders as well as in chronically catheterized swimbladders. No air-breathing was observed under hyperoxia in our experiments, therefore, the oxygen must have been taken up via the gills and transferred to other organs, including the posterior swimbladder. The posterior bladder has a rich vascularization and thus blood supply, whereas the anterior swimbladder is poorly vascularized. This together with the higher PO_2_ value recorded in the anterior bladder under normoxic conditions explain why the oxygen content in the anterior bladder was not yet significantly elevated under hyperoxic conditions.

In contrast to our previous study, where air breathing continued during hyperoxia (Pelster et al. 2016), in the present study, no air-breathing activity was observed in jeju under hyperoxic conditions. A significant reduction in air-breathing frequency under hyperoxic conditions has also been reported by Farrell and Randall (Farrell and Randall 1978). The most likely explanation for this difference appears to be the temperature. Our previous study has been performed on board a ship with temperatures of 30–35 °C (Pelster et al. 2016), while the study of Farrell and Randall and this study have been performed at temperatures between 25 and 28 °C. The higher temperature significantly stimulates metabolic activity and oxygen requirements, but the solubility of oxygen in water decreases with temperature, impairing aquatic oxygen uptake via the gills. It is, therefore, conceivable that at high temperatures jeju resort to aerial respiration even under hyperoxic conditions to supplement oxygen uptake.

Interestingly, under severe hypoxia in traira, almost no activity was detectable, and even ventilatory activity was often hard to detect. This again contrasts with our previous study performed at a higher temperature, where traira started to perform aquatic surface respiration during severe hypoxia (Pelster et al. 2016). The present observations suggest that at 26 ± 1 °C, traira adopted a severe depression of metabolic activity to withstand the hypoxic period. Occasionally, this behavior was also seen in jeju, suggesting that the jeju has two options to survive under hypoxia, either resorting to intensive air breathing, or reducing activity and referring to metabolic depression, as does the traira.

### Flexibility of the ROS defense system

The comparison of the ROS defense capacity of the jeju and the traira confirmed our previous study revealing a better ROS defense capacity in the facultative air-breathing jeju (Pelster et al. 2016), although the differences were not as clear as in the previous study. In the present study, catalase activity was even slightly higher in the traira as compared to jeju. Nevertheless, SOD and GR activity and also the concentration of GSH + GSSG were higher in jeju under control conditions. In addition, GR and GPx activity and GSH + GSSG concentration overall were higher in jeju as compared to traira. This confirms our previous observation that the glutathione-based ROS defense system appears more important in jeju than immediate ROS breakdown by SOD (Pelster et al. 2016). The differences in the responses in comparison to our previous study most likely appear to be attributable to the lower temperature used for the present experiments. ROS formation is temperature dependent and increases with higher temperatures. Therefore, the variable oxygen tensions encountered with an enhanced air-breathing activity may stimulate the expression of a well-organized ROS defense system in the jeju, especially at higher temperature.

Considering the flexibility of the ROS defense system, our results revealed that 4 h of hypoxia or hyperoxia were sufficient to induce changes in some of the ROS-degrading enzymes. The responsiveness observed in traira by far exceeded the responsiveness detected in jeju swimbladder tissue. While in the jeju an elevation of activity was only observed for GPx under hyperoxic and hypoxic conditions, in traira, in turn, activities of SOD, GR and GPx were increased during hyperoxia, but catalase was reduced under hypoxia. This was surprising because our data demonstrated that the traira swimbladder plays no role in gas exchange, and the oxygen content was constant under all experimental conditions tested. Changing oxygen availability is detected by oxygen receptors located in the gills, and transmitted to the central nervous system (Lopes et al. 2010; Zachar and Jonz 2012; Milsom 2012). From there, the stimulus to modify the ROS defense capacity must reach various organs in the whole body; conceivably, the signal could be altered blood O_2_ or ROS levels, and/or other blood-borne or neural pathways. Previous studies have demonstrated that a modification of the ROS defense system is not only detectable in the gills but also in muscle tissue or in the liver, organs not in direct contact with the environmental water (see Introduction). Thus, the signaling cascade also included the swimbladder in traira.

For the jeju variable oxygen content in the bladder and thus in blood are frequently expected, depending on the air-breathing activity, and we indeed observed a significant scatter in the oxygen content of the swimbladder under normoxic conditions. Accordingly, the ROS defense system is probably pre-adjusted to variable oxygen concentrations. This may explain why in our experiments we did not observe a marked response in the ROS-detoxifying systems of swimbladders in animals exposed to either hypoxia or hyperoxia; only GPx activity was elevated in hyperoxic animals. In line with the increase in GPx activity during hyperoxia, total glutathione concentration was elevated in hyperoxia.

While our data clearly show responses of the ROS defense system to changing oxygen concentration in the swimbladder, experimental deflation of the swimbladder of *Opsanus tau* with subsequent increase in swimbladder oxygen content did not result in a change in enzyme activities of the ROS defense system (Morris and Albright 1984). In the European eel, however, the process of silvering, which is the preparation for the deep seawater spawning migration, is connected to a significant improvement of the ROS defense system (Schneebauer et al. 2016). Very probably, this is because much higher oxygen partial pressures are expected to occur in the swimbladder during exposure to elevated hydrostatic pressure during spawning migration than during freshwater residence (Pelster 2015). The response of the ROS defense system to changing oxygen availability appears to be quite variable and species specific. Juvenile *Oncorhynchus mykiss* exposed for 4 h to hyperoxic conditions (> 40 mg O_2_ L^− 1^) show an elevated catalase activity in gills and liver 24 h after the exposure, but not at 1 to 12 h. SOD, total GPx and GR activity, however, were not affected (Ritola et al. 2002a). A very rapid response was observed in red blood cells, which showed an increase in SOD and catalase activity after only 5 min of exposure to hyperoxia (Ritola et al. 2002b). In liver of Atlantic salmon, 140–150% oxygen saturation for several weeks increased SOD and catalase activities (Lygren et al. 2000) but in goldfish, most enzyme activities tested were not changed during hyperoxic exposure of 12 h (Lushchak et al. 2005a).

Goldfish exposed to anoxic conditions increased catalase activity in liver, GPx and G6PDH activity in brain (Lushchak et al. 2001) and hypoxia increased SOD activity in liver, brain and gills in common carp (Vig and Nemcsok 1989), and catalase and GPx activity in brain (Lushchak et al. 2005b). Analysis of the hypoxia response of epaulette shark, threespine stickleback and rainbow trout combined with a literature survey looking at 13 species of fish and one shark revealed that fish in general do not show an increase in antioxidant defense response under hypoxia, and if a response is seen, it is not related to the hypoxia tolerance of the species (Leveelahti et al. 2014). Following reoxygenation after a hypoxic episode, often an enhancement of the ROS defense capacity is observed, which is discussed as a preparation for the oxidative stress encountered during recovery from hypoxia (Hermes-Lima et al. 1998; Lushchak and Bagnyukova 2006; Welker et al. 2013).

The present study revealed significant differences in physiology and performance of the swimbladder of two erythrinid fish. The use of the swimbladder as an air-breathing organ in the jeju results in highly variable PO_2_ values in the swimbladder, depending on the frequency of breathing air. Swimbladder PO_2_ also appeared to be related to blood flow to the swimbladder, as suggested by the rapid increase in PO_2_ (within 30 min) under hyperoxic conditions when no air breaths were taken, and the high variability of bladder PO_2_ at a similar number of breaths taken under normoxic conditions. The high variability of swimbladder PO_2_ coincided with an improved ROS defense system, as compared to swimbladder tissue not used for aerial respiration. In consequence, hypoxic or hyperoxic conditions did not have a strong influence on the capacity of the ROS defense system. In the traira, which uses the swimbladder as a buoyancy organ and not for breathing air, swimbladder PO_2_ values remained constant, irrespective of environmental PO_2_. Changing environmental PO_2_ values, however, caused a much stronger response in the ROS defense system than observed in jeju. This indicated that changes in environmental PO_2_ may influence the ROS defense system not only in tissues immediately exposed to the varying PO_2_ but also in organs not directly confronted with the modified PO_2_.
